# Solvent-Free Synthesis of P(MMA-AA) Copolymers and Their Application as Sustainable Primers for Concrete Substrates

**DOI:** 10.3390/polym17223039

**Published:** 2025-11-17

**Authors:** Aisha Nurlybayeva, Zhansaya Urkimbayeva, Raikhan Rakhmetullayeva, Raushan Taubayeva, Nurbanu Sarova, Ainur Seitkan, Gulnaziya Seitbekova, Kamila Bulekbayeva, Bakytgul Kussainova, Assem Shinibekova, Rustem Ergali

**Affiliations:** 1 Taraz University Named After M. Dulaty, Taraz 080000, Kazakhstan; rustem_ergali@mail.ru (A.N.); raushan.taubaeva@mail.ru (R.T.); nurhat2000@mail.ru (K.B.); aa.shinibekova@dulaty.kz (A.S.); 2 Al-Farabi Kazakh National University, Almaty 050010, Kazakhstan; 5858992@gmail.com (Z.U.); raikhan.rakhmetullayeva@gmail.com (R.R.); 3 Kazakh National Medical University Named After S.D., Asfendiyarov, Almaty 050010, Kazakhstan; 4 Higher School of Natural Sciences, Astana International University, Astana 010000, Kazakhstan; seitkanainur.77@mail.ru; 5 M. Utemisov West Kazakhstan University, Uralsk 090010, Kazakhstan; nurbol-bakytgul@mail.ru; 6 Department of Architecture and Construction Production, Kazakh National University of Water Management and Irrigation, Taraz 080000, Kazakhstan; rustem-ergali79@mail.ru

**Keywords:** methyl methacrylate, acrylic acid, copolymerization, solvent-free coatings, mechanical properties, thermal stability, wettability, crosslinking density

## Abstract

This study reports the solvent-free synthesis, structural characterization, and performance evaluation of poly (methyl methacrylate-co-acrylic acid) [P(MMA-AA)] copolymers intended for use as sustainable concrete primers and industrial coatings. A series of copolymers with varying MMA-AA molar ratios were synthesized via bulk radical polymerization and characterized using ^1^H and ^13^C nuclear magnetic resonance (NMR) spectroscopy, thermogravimetric analysis (TGA), and differential scanning calorimetry (DSC). The resulting materials were evaluated for their physical mechanical properties, including viscosity, tensile strength, surface hardness, and wettability. The findings revealed that higher MMA content improved the thermal stability, tensile strength, and hardness of the coatings, whereas increasing the AA content enhanced crosslinking density, hydrophilicity, and chemical resistance. These results demonstrate the potential of solvent-free P(MMA-AA) copolymers as environmentally friendly, high-performance alternatives to conventional solvent-based systems in protective coating applications.

## 1. Introduction

The growing need for environmentally friendly building materials has attracted attention to the development of low-emission concrete coatings. Traditional solvent-based coatings have excellent mechanical and protective properties but at the same time emit large amounts of volatile organic compounds (VOCs) [1,2] that are harmful to the environment and human health [3,4]. Polymer coatings based on acrylate and methacrylic acid structures have become promising options due to their enhanced adaptability, mechanical strength, and chemical resistance [5,6,7].

Methyl methacrylate (MMA) and acrylic acid (AA) are widely used monomers for creating functional copolymers in many fields, including construction, biomedicine, and packaging. Their copolymerization makes it possible to obtain more hydrophilic, viscous, and flexible materials; therefore, they can be used as primers for concrete [8,9]. Nevertheless, most polymer coatings available on the market are still based on organic solvents or water-based systems, which negatively affect both performance and environmental sustainability. Water-based paints are more environmentally friendly, but problems such as mold growth, cracking, poor adhesion, and brittleness often occur during the cold season [10,11].

To overcome these limitations, solvent-free polymer systems are being considered as innovative concrete coatings. Acrylate and methacrylic acid copolymers have many advantages, including the ability to be produced without solvents, better adhesion to cement-based surfaces, and adjustable mechanical properties. In addition, these materials comply with the principles of green chemistry and the circular economy due to their recyclability and ability to reduce environmental impact [12,13].

Previous research has focused on copolymers synthesized from MMA and AA, mainly for aqueous or biomedical applications. Gyurova et al. [14] investigated the properties of MMA-AA copolymers in aqueous solutions and the role of acrylic acid in increasing solubility and ionization. However, the mechanical properties and structural performance of solvent-free MMA-AA copolymer films have not been sufficiently studied. Similarly, Shi et al. [15] examined the tensile properties of starch films modified with methacrylic acid but did not consider their use as primers for building materials.

The synthesis and comprehensive characterization of solvent-free P(MMA-AA) copolymers with varying MMA/AA molar ratios were performed to evaluate their effectiveness as high-performance concrete primers, particularly in low-temperature conditions [16,17]. The copolymers were produced via bulk radical polymerization, a method frequently employed for solvent-free acrylic systems [6,7]. Their characterization was performed utilizing ^1^H and ^13^C NMR spectroscopy, thermogravimetric analysis (TGA), and differential scanning calorimetry (DSC) [18,19,20]. We systematically examined their mechanical properties, encompassing compressive, tensile, and flexural strength, in addition to curing time and pot life. An augmentation in acrylic acid concentration enhanced the coating’s durability and longevity, substantiating that these materials may serve as enduring and efficient means of safeguarding concrete in frigid environments [17,21].

A novel method for synthesizing P(MMA-AA) copolymers without solvents was employed, ensuring that the materials were environmentally friendly and effective. Azobisisobutyronitrile (AIBN) initiated the copolymerization of methyl methacrylate (MMA) and acrylic acid (AA) as a radical initiator. The reaction mixture gradually transformed into a viscous, gel-like substance following the addition of the initiator. The product was refined by dissolving it in acetone and subsequently precipitating it with hexane, resulting in a solid copolymer. The purified copolymer was reintroduced into its monomer, methyl methacrylate, without the incorporation of additional organic solvents. This created a homogeneous liquid system suitable for coating [8,9].

**Figure 1:**
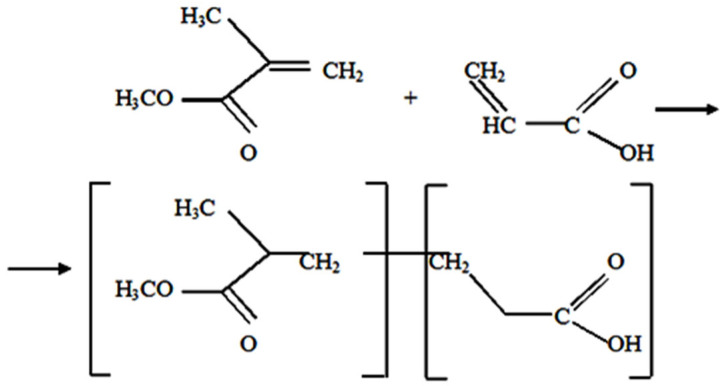
Reaction mechanism of copolymer synthesis.

This technique is an innovative use of bulk radical polymerization for the synthesis of MMA-AA copolymers without the necessity of solvents. It integrates the principles of green chemistry with the development of functional materials intended for application as concrete primers. Figure 1 illustrates a schematic representation of the radical copolymerization process, resulting in a random P(MMA-AA) copolymer composed of alternating units of methyl methacrylate (MMA) and acrylic acid (AA). Polymerization occurs when the double bonds in the monomers are cleaved. Subsequently, chain propagation occurs, resulting in a structure whereby MMA units confer strength and thermal resistance, while AA units impart hydrophilicity and interfacial adhesion.

## 2. Materials and Methods

### 2.1. Materials

Methyl methacrylate (MMA, ≥99%, Sigma-Aldrich, St. Louis, MO, USA), acrylic acid (AA, ≥99%, Sigma-Aldrich, St. Louis, MO, USA), 2,2′-azobisisobutyronitrile (AIBN, 98%, Sigma-Aldrich, St. Louis, MO, USA), N,N-dimethyl-p-toluidine (DMPT, 98%, Sigma-Aldrich, St. Louis, MO, USA), hydroquinone (HQ, 99%, Sigma-Aldrich, St. Louis, MO, USA), and benzoyl peroxide (BPO, 98%, Sigma-Aldrich, St. Louis, MO, USA) were used as received. Acetone and hexane (analytical grade, Sigma-Aldrich, St. Louis, MO, USA) were used for the precipitation and washing of the copolymers. Deuterated chloroform (CDCl_3_) and deuterium oxide (D_2_O) (≥99.8 atom % D, Sigma-Aldrich, St. Louis, MO, USA) were used as solvents for NMR spectroscopy. All reagents were used without further purification unless otherwise specified.

### 2.2. Synthesis of Copolymers of Methyl Methacrylate and Acrylic Acid

**Figure 2:**
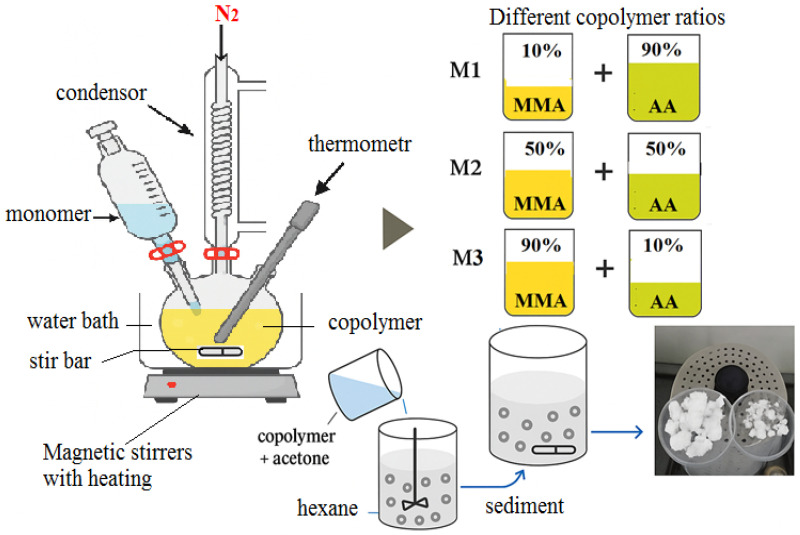
Synthesis of methyl methacrylate and acrylic acid copolymers.

The synthesis of the copolymer was carried out on a special installation. A three-necked round-bottomed flask with a capacity of 500 mL, equipped with a mechanical stirrer and a dropping funnel, was placed in a water heater, gaseous nitrogen was introduced through a special tube, and the internal temperature was monitored with a thermometer. A three-neck round-bottom flask is connected to the condenser. The water inlet and outlet hoses are connected to the condenser. The installation for such synthesis is shown in Figure 2.

A copolymer based on these two monomers was synthesized by mass polymerization according to the mechanism of radical polymerization in the presence of the initiator 2,2-azo-bis-isobutyronitrile (AIBN). The initial composition of the monomer mixture was obtained at the following volume ratios: MMA-AA = 10-90 (M1), 50-50 (M2) and 90-10 (M3) mol.%.

In the synthesis process, a mixture of MMA and AA monomers (20 mL) is poured into a round-bottom three-neck flask equipped with a mechanical stirrer, a dropping funnel and a water bath, and thoroughly mixed until the heating temperature reaches 70 °C and gaseous nitrogen or argon is gradually supplied. The flask was then stirred evenly by dropping a mixture of the remaining MMA and AA monomers (80 mL) with initiating agent AIBN (0.1 g) into a three-necked flask for 40 min, after which the temperature was raised to 80 °C and the synthesis process was allowed to proceed by 3 h. The role of temperature and mechanical mixing is of particular importance due to the specifics of bulk polymerization. At the end of the synthesis, after lowering the temperature of the reaction mixture to 20 °C, the resulting suspension is first dissolved in acetone and then precipitated in hexane. After, it was dried in air until a stable weight was reached, and then dried in a vacuum oven [22].

### 2.3. Primer Film Formation

The copolymer syrup was prepared by dissolving the copolymer (MMA-AA) and benzoyl peroxide in MMA monomer at normal temperature (25 °C). Then, a liquid component was prepared using MMA monomer, DMPT, and hydroquinone. Benzoyl peroxide and DMPT were added to the syrup at a concentration of 1.5 and 0.75 parts per hundred (pph) as an initiator and accelerator, respectively. Hydroquinone was added to the syrup as an inhibitor. Paraffin wax was added at a rate of 1% of the syrup weight. Then, this copolymer syrup was added to the liquid component mixed at a ratio of 10 g copolymer/90 g methyl methacrylate, 15 g copolymer/85 g methyl methacrylate, and 25 g copolymer/75 g methyl methacrylate to maintain the mixing ratio at 100% as shown in Table 1.

Acryl syrups were mixed manually with calcium carbonate, sand and pigment in room temperature. The viscosity of mixtures increases noticeably in few minutes due to the viscosity of acryl syrups. Adding to the mixture BO and mixed slowly for 2 min followed by adding DMPT. When the dough state was reached, the pastes were poured and placed in the glass mold with size 200 mm × 110 mm × 3 mm and allowed to cure in room temperature. Details of mixes are given in Table 2.

### 2.4. Characterization of Materials and Analytical Methods

The ^1^H NMR spectra of the prepared copolymers were recorded using a JEOL EX-270 NMR spectrometer (JEOL Ltd., Tokyo, Japan) operating at 270 MHz at room temperature with a superconducting magnet and a 5 mm dual probe head for ^1^H. The spectral width was set to 1/4 and 4000 Hz for the proton signal. Thermogravimetric analysis (TGA) was carried out using a TGA/SDTA851e analyzer (Mettler Toledo, Greifensee, Switzerland) according to ISO 11358-1 [23]. Pot life and curing time tests were performed according to DIN EN ISO 9514 and ASTM D5895-03 [24,25], respectively. Once the prepared primer mixture formed a viscous mass, it was poured into a 200 mm × 110 mm × 3 mm glass mold and dried at room temperature. For compressive strength tests, cubic specimens (5 × 5 × 5 cm) were prepared in accordance with KS F 2419 [26] and cured at 200 °C. The compressive strength of the floor primer was determined according to KS F 2405 [27] using a Pilot-4 universal testing machine in compression mode at a load rate of 2 mm/min (Controls Group, Liscate, Italy).

Water absorption tests were carried out in accordance with BS 1881-122:2011 (Concrete) [28]. Dynamic surface wettability was evaluated using a CDCA-100F dynamic adsorption analyzer (Camtel, Essex, UK) in accordance with BS 1881-122:2011 [28]. Each sample (1 × 5 cm) was immersed in distilled water for six months, and the absorbed water mass was measured using a CDCA-100F dynamic adsorption analyzer (Camtel, Essex, UK) in accordance with BS 1881-122:2011 (Concrete).

The measured water absorption ranged from 0.0038 mg (F-Syr10/M1) to 0.0005 mg (F-Syr25/M2), confirming the excellent moisture resistance of the MMA-AA primers.

These results are consistent with literature data for PMMA-based acrylic copolymers, which show similarly low equilibrium water sorption even after prolonged immersion [29,30]. The abrasion resistance of the coatings was determined according to ASTM D2688-94 [31].

## 3. Results and Discussion

### 3.1. ^1^H and ^13^C NMR Spectroscopy

**Figure 3:**
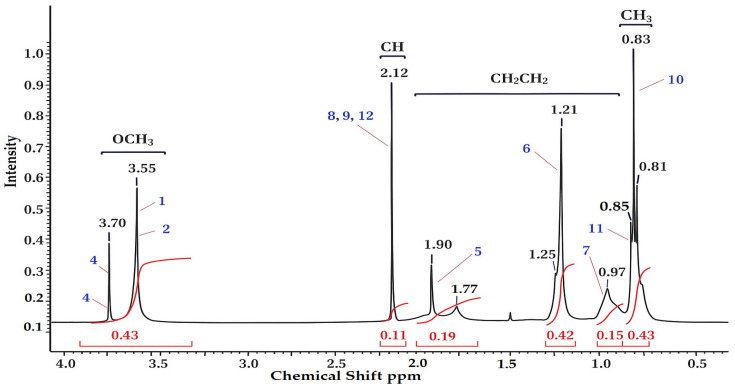
NMR (^1^H) spectrum of MMA-AA 10-90 (M1) mol. % copolymer.

**Figure 4:**
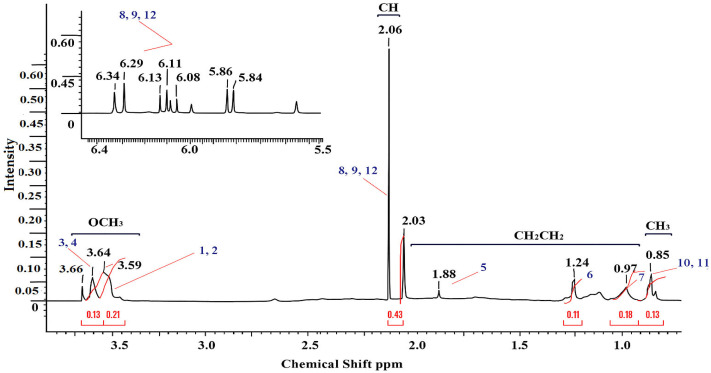
NMR (^1^H) spectrum of MMA-AA 50-50 (M2) mol. % copolymer.

**Figure 5:**
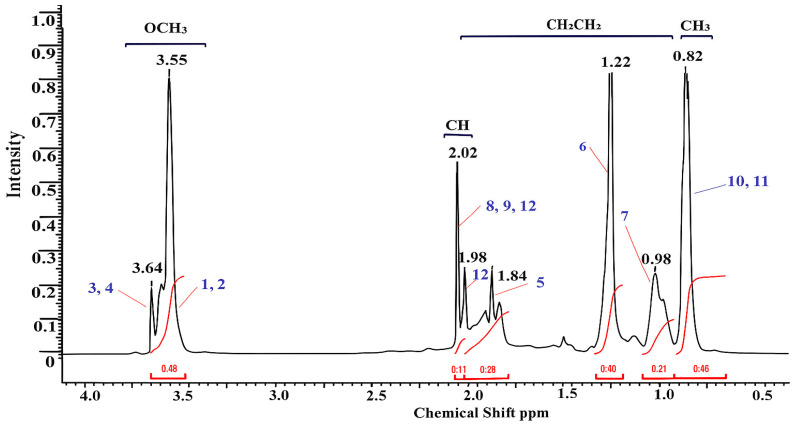
NMR (^1^H) spectrum of copolymer MMA-AA 90-10 (M3) mol. %.

The copolymer based on MMA-AA was analyzed by proton NMR ^1^H and ^13^C method. The structural formula of the copolymer was determined based on the signals of the functional groups, taking spectra for water-soluble MMA and AA-based copolymers, in the solvent D_2_O (deuterium) in the range of 300–400 MHz. The peaks of chemical shift in the proton spectrum of MMA monomer units (Figure 3, Figure 4 and Figure 5) belong to the group of 0.83–0.85 ppm methyl (CH_3_) and 3.55–3.64 ppm methoxyl (OCH_3_). In the acrylic acid monomer, the proton of the carbon atom was recorded at a frequency of 2.02–2.12 ppm. Peaks of methylene and (COO–) carboxyl groups were detected in the region of 0.97–1.90 ppm in the copolymer link [18,19]. The oscillation bands characteristic of the functional groups of the copolymer are shown in Table 3.

Using the normalized values of the integral intensities of the ^1^H signals it is possible to calculate the molar ratio of monomer units in the MMA and AA copolymer (Equations (1)–(3)).

(1) $$I\left({CH}_{3\left(MMA\right)}\right):I\left({CH}_{\left(AA\right)}\right)=\frac{0.43}{3}:\frac{0.11}{1}=0.14:0.11=1.27:1,\;\;\;M1\left(10:90\right)$$

(2) $$I\left({CH}_{3\left(MMA\right)}\right):I\left({CH}_{\left(AA\right)}\right)=\frac{0.13}{3}:\frac{0.43}{1}=0.43:0.43=1:1,\;\;\;M2\left(50:50\right)$$

(3) $$I\left({CH}_{3\left(MMA\right)}\right):I\left({CH}_{\left(AA\right)}\right)=\frac{0.40}{3}:\frac{0.11}{1}=0.13:0.11=1.18:1,\;\;\;M3\left(90:10\right)$$

In the carbon spectrum, the signals of the MMA link are observed at 14.13 (CH_3_) and 51.85 ppm (OCH_3_). The characteristic signal of the methine carbon atom of AA appears in the region of 31.62 ppm. At frequencies 18.36, 22.68 and 30.93 ppm resonation of the CH_2_ groups of the monomer units is assumed (Table 4). The most downfield signals (176.93 and 178.15 ppm) can be attributed to carboxyl carbon atoms [32].

The ^13^C NMR spectra reveal signals indicative of both polymer units and residual monomers. Particular emphasis is placed on the pronounced signal at 31.6 ppm associated with the methyl carbon (–CH=), which typically vanishes post-polymerization. The severity signifies the existence of partially reacted acrylic acid or pre-polymerization byproducts. Carbonyl signals between 176 and 178 ppm indicate the existence of functional groups –COOH and –COOR, characteristic of copolymers composed of acrylic acid and methyl methacrylate. The signals between 44 and 52 ppm correspond to the quaternary and methoxyl carbons of the MMA unit, so confirming the incorporation of methyl methacrylate fragments in the structure. The analysis of ^13^C NMR spectra requires revision, as a prominent signal at 31.6 ppm signifies the presence of monomeric residues. This may signify incomplete polymerization. Simultaneously, carbonyl signals with ranges of 44–52 ppm corroborate the partial establishment of polymeric links between the MMA and AK units.

**Figure 6:**
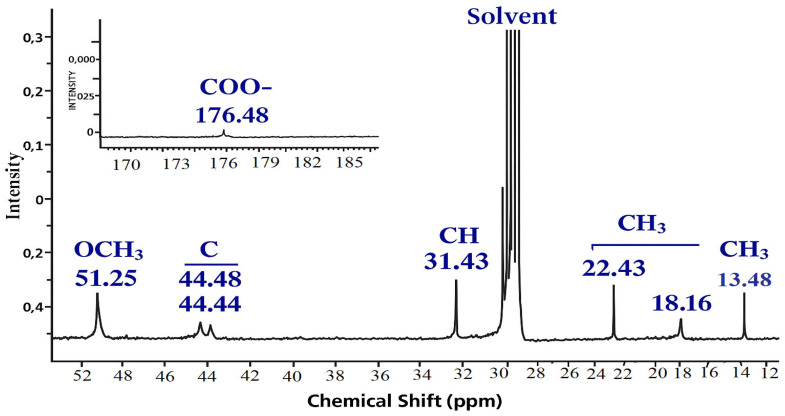
^13^C NMR spectrum of copolymer MMA-AA 50-50 (M2) mol. %.

**Figure 7:**
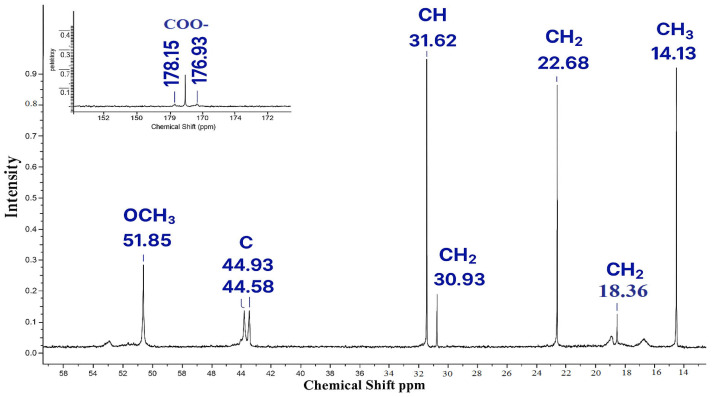
^13^C NMR spectrum of copolymer MMA-AA 90-10 (M3) mol. %.

**Figure 8:**
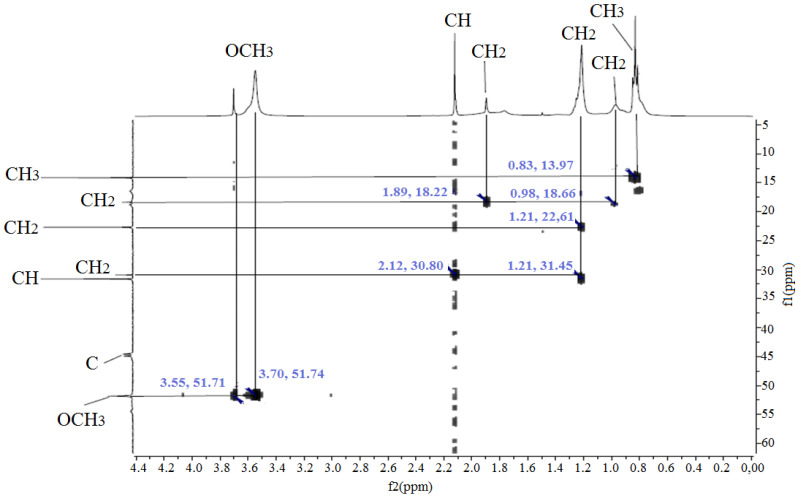
HMQC (^1^H-^13^C) NMR spectrum of copolymer MMA-AA 50-50 (M2) mol. %.

**Figure 9:**
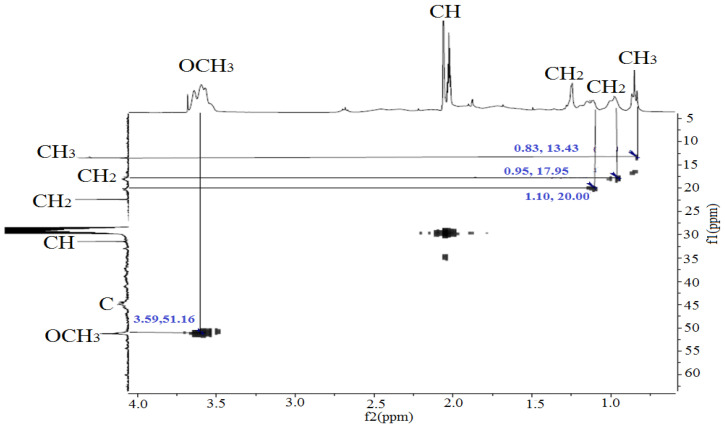
HMQC (^1^H-^13^C) NMR spectrum of copolymer MMA-AA 90-10 (M3) mol. %.

The correlation of the ^1^H and ^13^C resonance lines was confirmed by the two-dimensional HMQC spectrum (Figure 6, Figure 7, Figure 8 and Figure 9).

In the carbon spectrum, signals of the MMA link are observed at 13.48 (CH_3_) and 51.25 ppm (OCH_3_). The characteristic signal of the methine carbon atom of AA appears in the region of 31.45 ppm. At frequencies of 18.16 and 22.43 ppm resonation of the CH_2_ groups of the monomer units is assumed [33]. To verify the chemical structure of the copolymers, the successful copolymerization of methyl methacrylate (MMA) and acrylic acid (AA) monomers is confirmed, and the monomer structure and group distribution in the polymer chain are evaluated. It is important to understand which functional groups are present in these polymers and how they can affect the adhesion to the concrete surface (e.g., the presence of –COOH groups).

### 3.2. Thermal Analysis

Thermogravimetric analysis (TGA) was used to assess the thermal stability and decomposition behavior of the synthesized MMA-AA copolymers. It provides important information about the decomposition stages, onset temperatures, and amount of residual carbon, allowing for a comparative assessment of the influence of monomer composition on the thermal behavior of the copolymers.

As can be clearly seen, ***T_g_*** varies continuously with the monomer concentration in the copolymer. The so-called Fox equation is used to describe the dependence of ***T_g_*** on this phenomenon (Equation (4)):

(4) $$\frac{1}{T_{g}}=\frac{w_{1}}{T_{g1}}+\frac{w_{2}}{T_{g2}}$$

where ***T_g_*** is the glass transition temperature of the copolymer, ***T_g_***_1_ and ***T_g_***_2_ are the glass transition temperatures of the three homopolymers, and ***w***_1_ and ***w***_2_ are the weights of the three repeat copolymers (Table 5) [34].

The experimentally measured ***T_g_*** values are close to the predicted ones based on Fox equation. Generally, it is known that ***T_g_*** is directly proportional to cross-linking density and indirectly proportional to chain flexibility. Results are consistent with this statement.

The Fox equation was used to determine the glass transition temperature ***T_g_*** of copolymers MMA-AA, the characteristic parameters are shown in Table 5.

The thermal stability of the synthesized copolymers and composite sorbents was evaluated using a TGA/SDTA 851e analyzer (Mettler Toledo, Greifensee, Switzerland). Samples weighing 10–15 mg were heated from room temperature to 850 °C at a constant rate of 20 °C min^−1^ under a nitrogen atmosphere with a gas flow rate of 30 mL min^−1^. The mass loss profiles were recorded as a function of temperature, and the derivative thermogravimetric (DTG) curves were used to determine characteristic decomposition stages and thermal transition points.

**Figure 10:**
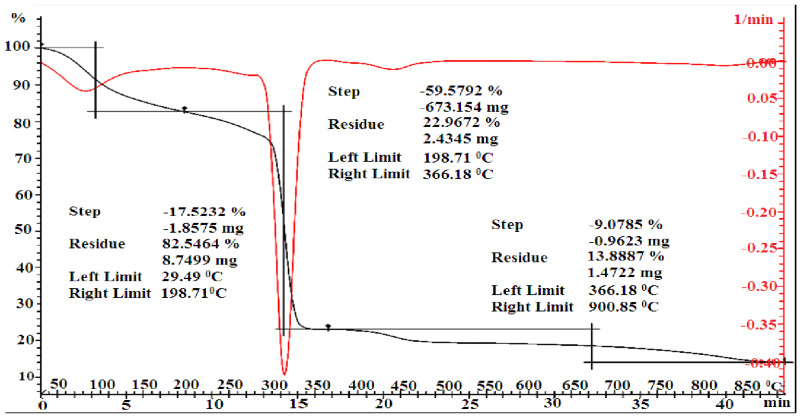
Thermogravimetric analysis of copolymers based on MMA-AK 10-90 (M1) mol. %.

**Figure 11:**
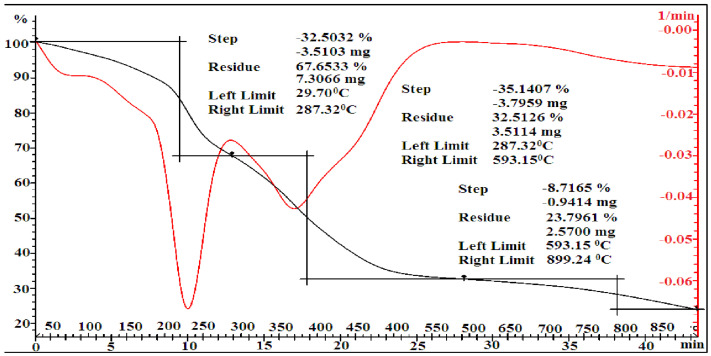
Thermogravimetric analysis of copolymers based on MMA-AK 50-50 (M2) mol. %.

**Figure 12:**
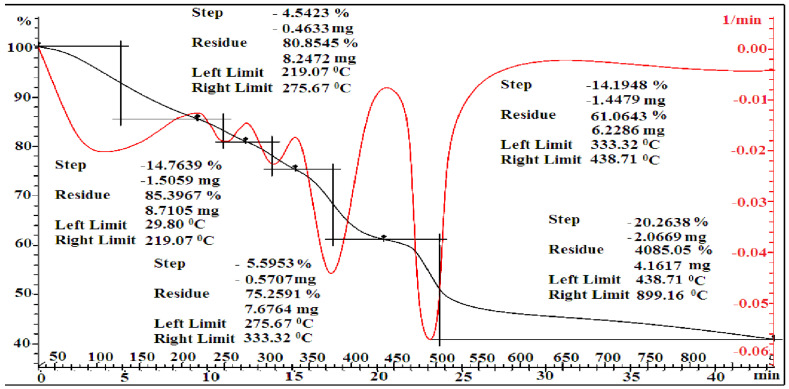
Thermogravimetric analysis of copolymers based on MMA-AK 90-10 (M1) mol. %.

According to the data of TGA, the decomposition stages of MMA-AA copolymer samples in the ratio 10-90 (M1), 50-50 (M2) and 90-10 (M3) are shown in Figure 10, Figure 11 and Figure 12. It can be observed that the decomposition stages of the copolymer 90-10 (M3) consist of 5 stages.

The decomposition of the copolymer MMA-AA in several stages is due to the inhibition of the movement of the macromolecule of the copolymer caused by intermolecular interactions between the monomer units of MMA and AA, resulting in weight loss by water loss and the binding of copolymer components. In the first and second stages, when the composition of the copolymer MMA-AA is 10-90, a mass of 17.53–59.57% is lost in the temperature range 29.49–366.18 °C, and when the composition is 50-50, in the temperature range 29.70–593.15 °C, the mass loses 32.50–35.14%. Loses of mass of 14.76–4.54% are observed in the temperature range 29.8–275.67 °C in the 90-10 ratio.

The initial and secondary stages of weight loss are attributed to the evaporation of chemically bound water and residual solvents.

In the tertiary stage, weight loss was observed within the temperature range of 366.18–900.85 °C for the 10-90 MMA-AA copolymer, 593.15–899.24 °C for the 50-50 ratio, and 275.67–333.32 °C for the 90-10 ratio. The thermal degradation of the copolymer P (MMA-AA) (90-10, M3) transpires in five principal stages, as seen in Figure 6. During the initial phase, the physically adsorbed moisture evaporates within the temperature range of 29.80–219.07 °C, leading to the elimination of low molecular weight volatiles and a mass loss of 14.76%. During the second stage, the primary chain of carboxyl groups is degraded, resulting in the release of CO_2_ and H_2_O at temperatures between 219 and 276 °C. During the third phase, at temperatures ranging from 276 to 333 °C, the ester linkages dissociate, resulting in the degradation of polymethyl methacrylate. Subsequently, further weight reduction for 90-10 copolymer content transpires during the fourth and fifth stages, within the temperature intervals of 333.32–438.71 °C and 438.71–899.16 °C. This mass loss is attributed to the exothermic decomposition of the COOH group, leading to the disruption of internal and intermolecular interactions due to the hydrogen bonding between MMA and AA. This analysis was employed to assess the thermal stability of the synthesized copolymers, which is crucial for materials used in construction that must retain their properties under service conditions, such as the heating of concrete surfaces during summer.

The thermogravimetric analysis (TGA) results facilitated the determination of the onset of thermal degradation and the overall thermal behavior of the material. TGA was instrumental in assessing the thermal stability of the copolymers, which is essential for determining the solidification temperature and resistance to degradation. Collectively, these methodologies provide a comprehensive understanding of the structure-property relationships necessary for the development of effective Filmers for concrete [19,28].

### 3.3. Gel Permeation Chromatography

GPC provides a more convenient method for determining the molecular weights of polymers. In fact, most samples can be thoroughly analyzed in an hour or less [29,30]. Thus, GPC has allowed for a quick and relatively easy estimation of molecular weights and distribution for polymer samples.

**Figure 13:**
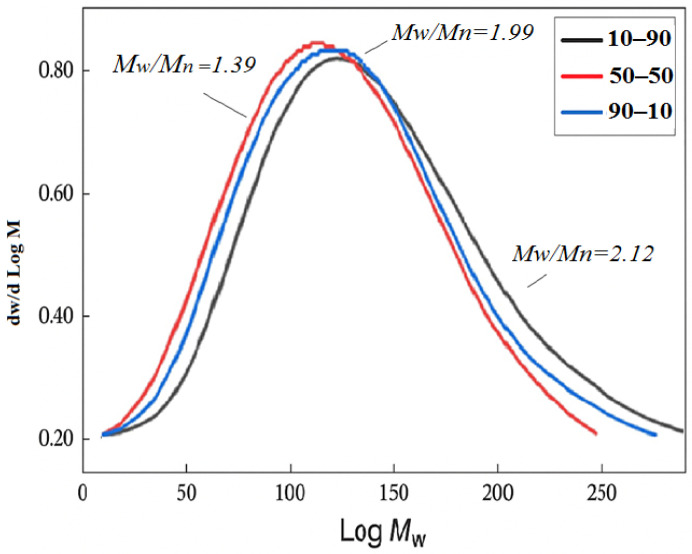
Distribution of molar masses for a sample of MMA-AA copolymer in different ratios 10-90, 50-50, 90-10.

**Figure 14:**
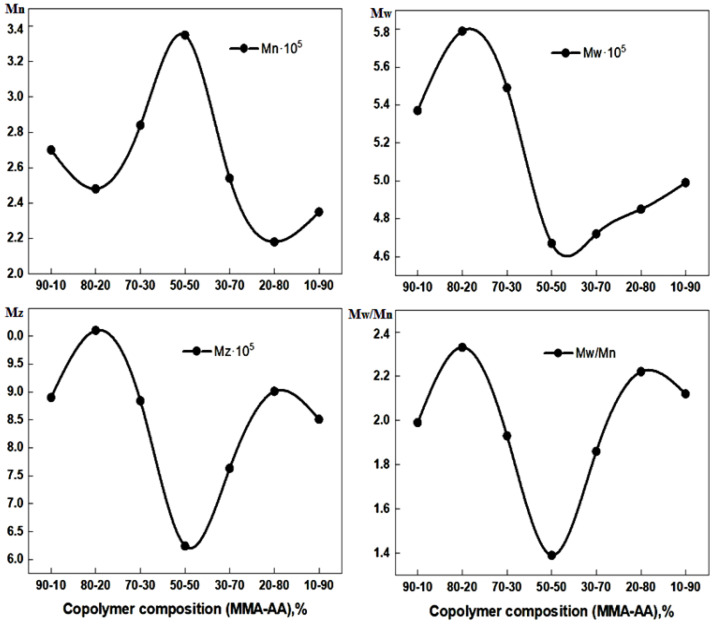
Molecular weight characteristics of MMA-AA copolymers Mn, Mw, Mz and Mw/Mn.

A number of MMA and AA copolymers of different compositions (90-10, 70-30, 50-50, 30-70 and 10-90) were synthesized by free radical polymerization and their molecular weight characteristics were studied by gel permeation chromatography (Table 6, Figure 13 and Figure 14).

It is evident that all copolymers have a sufficiently high molecular weight, the value of which depends little on the composition of the initial monomer mixture and, accordingly, for such copolymers. In addition, most of such MMA-AA copolymers are characterized by a low value of the polydispersity coefficient PDI, which indicates a fairly narrow molecular weight distribution of the obtained copolymers.

The 90-10 MMA-AA copolymer exhibits an average molecular weight (Mn) of 270,000, an average molecular weight (Mw) of 537,000, and a z-average molecular weight (Mz) of 890,000 with a polydispersity index (PDI) of 1.99. These results suggest a moderately broad molecular weight distribution typical of radical polymerization methods.

The high Mz value suggests the presence of a small fraction of very high molecular weight species, which may affect the mechanical and rheological properties of the copolymer. These results indicate that the molecular weight distribution and polydispersity index (PDI) are significantly dependent on the monomer composition.

The general trend shows that with increasing acrylic acid content, the molecular weight distribution changes. The polydispersity index (PDI) varies from 1.39 (for 50-50 MMA-AA) to 2.12 (for 10-90 MMA-AA), indicating differences in polymerization behavior. The highest Mn value (335,000) was observed for the 50-50 MMA-AA copolymer, suggesting optimal chain growth under these conditions. The changes in Mz suggest changes in the presence of high molecular weight fractions.

To assess the uniformity of the molecular weight distribution in different monomer compositions, the polydispersity index (Mw/Mn) of MMA-AA copolymers was evaluated. As shown in Figure 8, the index exhibits a U-shaped trend close to the least equimolar composition (50% AA). This indicates that free radical polymerization leads to the most uniform chain length at this ratio. In contrast, a broader molecular weight distribution is observed at low and high AA compositions (10–20% and 80–90%), which is associated with differences in reactivity coefficients and dispersion efficiency. Each of the above chemical methods allowed us to fully characterize the synthesized P(MMA-AA) copolymers and confirm their structural, thermal, and molecular suitability for use as Filmers for concrete substrates. These data justify the selection of the composition and scientifically substantiate the potential effectiveness of polymer Filmers in construction practice. Gel permeation chromatography (GPC) has been used to measure the molecular weight properties of copolymers, including weight average (Mw) and molecular weight (Mn), as well as the polydispersity index (Mw/Mn). These parameters are important for predicting the rheological properties, film-forming ability, and strength of the resulting materials. The stability and uniformity of molecular weight directly affect the uniformity and efficiency of precast concrete applications [4,31,32].

The findings from gel permeation chromatography (GPC) (Table 5, Figure 13 and Figure 14) demonstrate that the molecular weight characteristics of the P(MMA-AA) copolymers are significantly influenced by the monomer composition. The number-average (Mn) and weight-average (Mw) molecular weights reach their maximum values (Mn = 3.35 × 10^5^ and Mw = 4.67 × 10^5^) at the equimolar composition (50-50 MMA-AA), indicating optimal chain propagation and termination during free radical copolymerization. This suggests that, at this ratio, the polymer chains grow in the most uniform manner, resulting in the narrowest molecular weight distribution.

As the ratio of acrylic acid (AA) to methyl methacrylate (MMA) changes, both the number-average (Mn) and weight-average (Mw) molecular weights decrease, indicating shorter polymer chains. This behavior can be attributed to the differing reactivity ratios of MMA and AA, along with the increased likelihood of chain-transfer reactions in AA-rich compositions. The z-average molecular weight (Mz) follows a similar non-linear trend, reaching its minimum at the 50-50 composition and higher values at more asymmetric ratios (10-90 and 90-10). This suggests the presence of small fractions of very high molecular weight species in these systems.

The polydispersity index (PDI = Mw/Mn) ranges from 1.39 to 2.12, showing a U-shaped dependence on the monomer ratio. The 50-50 copolymer exhibits the lowest polydispersity (PDI = 1.39), indicating a more homogeneous structure, while broader distributions at 10-90 and 90-10 suggest a more heterogeneous copolymerization process. The molecular weight distribution curves (Figure 14) further confirm this trend: the equimolar copolymer shows a sharp, symmetrical peak, whereas the non-equimolar samples exhibit broader peaks extending toward higher molecular weights.

These findings confirm that the MMA-AA ratio directly affects the molecular weight distribution and polydispersity index, which in turn determine the rheological and film-forming properties of the copolymers—key parameters for their application as primer binders on concrete substrates.

### 3.4. Characterization of Primer Films

#### 3.4.1. Pot-Life and Curing Test

**Figure 15:**
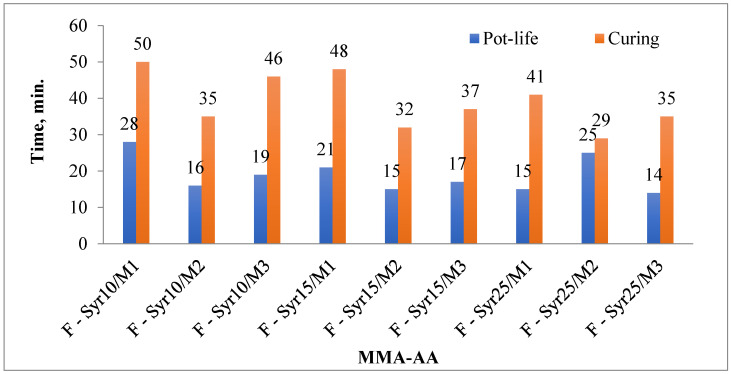
Pot life, curing time of primer films containing MMA-AA.

The durability and curing time of the floor coverings were studied, as shown in Figure 15. With the increase in the weight of MMA in the coating made from the MMA-AA copolymer, the durability and curing time of the primer decrease. This is due to the increase in the hard segment of MMA in the floor covering. On the other hand, the durability and curing time of the MMA-AA copolymer in the primer show very low values. In practice, the durability and curing time of the floor covering depend on the hard segment and the coupling agent in the primer.

Therefore, the durability and curing time of floor primers depend on the type of monomer and polymer used, the thickness of the primer layer, and environmental factors such as temperature and humidity [10,35,36,37,38,39]. The durability and curing time of coatings made from MMA-AA copolymers are relatively low. As can be seen from the graph, an increase in the MMA content results in a decrease in both durability and curing time. For example, the E-Cip25/M5 composition shows a pot life of 25 min and a curing time of 29 min, whereas for E-Cip25/M6, these values are reduced to 14 and 35 min, respectively. This behavior is attributed to the higher solids content of the primer and the structural characteristics of the copolymer.

These parameters are crucial for the practical application of flooring materials. A sufficient pot life is essential to ensure proper coating application, while the curing time should not be excessively long, as time efficiency is particularly important in production environments. Considering these two parameters (pot life and curing time) together allows for a comprehensive assessment of the technological and operational performance of the coating. A pot life that is too short can lead to difficulties during application, whereas a curing time that is too long may slow down the production cycle. Therefore, maintaining a balance between these two factors ensures the quality and effectiveness of the coating. Overall, the study results showed that both the service life and curing time of the coating decrease with increasing MMA-AA copolymer content. Taking this phenomenon into account is important when optimizing the formulation of flooring coatings. Moreover, these parameters are significantly influenced by factors such as the monomer and polymer type, coating thickness, temperature, and humidity [4,21,32,33,40].

#### 3.4.2. Compressive Strength

**Figure 16:**
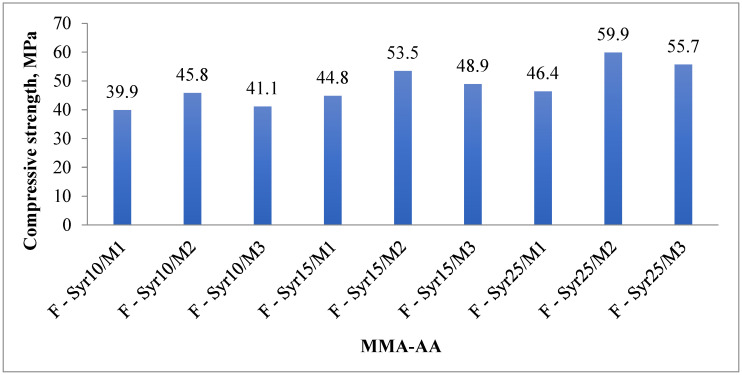
Compressive strength of copolymer primers with different ratios.

This study investigated the effect of MMA-AA copolymer syrup content with different compositions on the mechanical properties of primers, particularly their compressive strength. Floor primers containing MMA-AA copolymer syrup were used to determine the compressive strength of the samples, as shown in Figure 16. Generally, increasing the MMA content in the MMA-AA copolymer enhances the compressive strength of the primer. However, increasing the amount of copolymer in the acrylic syrup added to the floor primer decreases the compressive strength.

In the case of a 50:50 (MMA-AA) copolymer syrup ratio in the primer, the compressive strength was the highest among all groups: for example, the E-Syr10/M5 sample exhibited approximately 45.8 MPa, E-Syr15/M5 reached 53.5 MPa, and E-Syr25/M5 showed 59.9 MPa (Figure 16). The high compressive strength of the MMA-AA copolymer-based primer can be attributed to its low viscosity, high tensile strength, and hardness. In addition, the –COOH groups in the MMA-AA copolymer play a significant role in enhancing compressive strength, as the sand filler contains Si and Ca^2+^ ions, which form intermolecular complexes with CaCO_3_ [41].

Thus, the optimal monomer ratio in the MMA-AA copolymer is 50:50 with a syrup content of approximately 25%, ensuring maximum compressive strength (59.9 MPa). This composition can be recommended for use as a primer for concrete substrates subjected to high mechanical load requirements.

#### 3.4.3. Water Absorption Properties of the Primer

The results obtained in this study demonstrate the dynamic wetting properties of the primer in terms of water absorption. Based on these findings, the dynamic wetting behavior of the coating was analyzed through water absorption measurements. The mass of water absorbed after six months of immersion of the samples in water was determined. It was shown that the water absorption and weight loss of the MMA-AA copolymer syrup were significantly lower than those of the terpolymer and other MMA-AA based copolymer syrups used in the coating.

**Figure 17:**
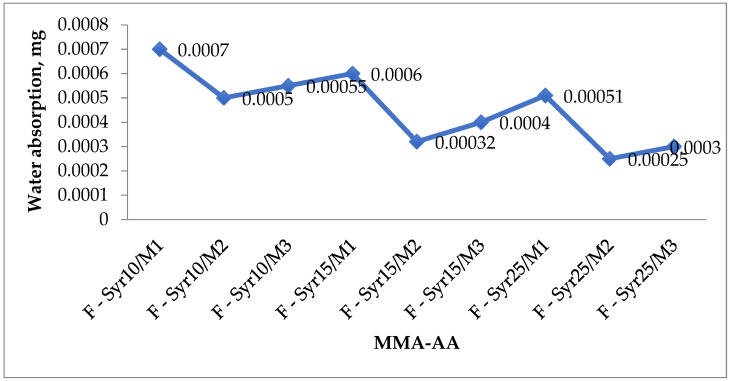
Water absorption characteristics of copolymer primers.

It was also found that as the number of –COOH groups in the acrylic syrup increases, the binder density in the floor coating rises, while the weight loss values decrease. A study of the chemical resistance of the acrylic coatings revealed that the coating prepared with the MMA-AA copolymer binder was durable and of high quality. The primer formulated with the MMA-AA copolymer syrup exhibited low water absorption (Figure 17). Moreover, with an increase in the MMA content in the acrylic syrup chains, the water absorption of the primer further decreased. Notably, the primer sample E-Syr25/M3 demonstrated the lowest water absorption values.

With an increase in the amount of MMA-AA copolymer in the acrylic syrup, the water absorption properties of the primer decrease. It was found that at a 25% amount of MMA-AA copolymer in the acrylic syrup, that is, the water absorption of the sample in group 3 (MMA-AA, 50-50), is significantly lower. Firstly, the decrease in water absorption properties is due to a smaller number of pores in the primer. This indicates a smaller number of porous spaces in the primer. However, the obtained results showed that the water absorption properties of the MMA-AA copolymer syrup contained in the primer were very low. As the proportion of methyl methacrylate (MMA) in the MMA-AA copolymer chains increases, water absorption decreases. Particularly low values were recorded for formulations containing 25% copolymer [16]. For example, samples of the F-Syr25 group exhibit minimal water absorption, reaching values of approximately 0.00025–0.0003 mg. The reduced water absorption is explained by a reduction in the number of pores and void spaces in the coating structure. This indicates a denser packing of the polymer matrix and improved intermolecular interactions between the copolymer chains and the mineral components of the primer. Thus, the introduction of MMA-AA copolymer in acrylic syrup significantly improves the moisture resistance of primer formulations. Application of the syrup at a 25% content is particularly effective, ensuring minimal water absorption.

#### 3.4.4. Weight Loss

**Figure 18:**
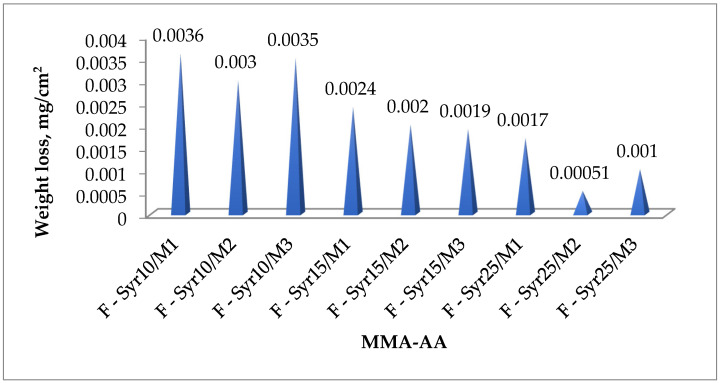
Weight loss of MMA-AA copolymer primer compositions.

Weight loss is the reduction in the initial mass of a material due to friction. This indicator reflects the ability of a coating to retain its shape and structure when exposed to erosion, as well as to withstand friction, cleaning, and other operational loads [34,35]. The results of the study (Figure 18) show that the introduction of MMA-AA copolymer syrup reduces coating weight loss. As the methyl methacrylate (MMA) content in the copolymer chains increases and the copolymer mass increases to 25%, the weight loss values decrease. This indicates an increase in friction resistance. The lowest weight loss was recorded for the F-Syr25/M5 composition (Group 3), indicating the highest resistance to abrasion and mechanical stress. Overall, a consistent pattern is observed: as the MMA monomer and copolymer content in the coating structure increases, weight loss decreases. One of the key factors determining friction resistance is the viscosity, strength, and other mechanical properties of the polymer matrix. Furthermore, increasing the number of carboxyl groups (–COOH) in the acrylic resin promotes an increase in the density of the primer-binder structure, which also leads to a reduction in weight loss. Thus, the use of MMA-AA copolymer syrup significantly improves the wear resistance of coatings, and the weight loss test method allows for an adequate assessment of friction resistance under conditions similar to actual use.

Research has shown that adding MMA-AA copolymer syrup to primer coatings significantly improves their performance properties (Table 7).

The optimal monomer ratio of 50:50 MMA-AA and 25% copolymer content ensure maximum compressive strength (59.9 MPa), minimal water absorption (0.00025–0.0003 mg), and minimal weight loss, indicating high wear resistance. Thus, the obtained results confirm the suitability of this composition for creating durable and mechanically strong primers for concrete substrates.

## 4. Conclusions

The use of acrylic polymers in paints as a binder is of particular interest for the development of industry and science. Therefore, for the first time in this work, copolymers based on methyl methacrylate and acrylic acid (MMA-AA) in various ratios were synthesized by free-radical copolymerization in bulk. The synthesized copolymers were used as a binder for solvent-free paints. These copolymers were obtained in the ratios of 10-90, 50-50, and 90-10. The weight of the copolymer was determined by gravimetric yield in the range of 86–87%. The structure of the copolymers based on synthesized acrylate and methacrylate was determined by NMR spectroscopy. The thermal analysis of the obtained copolymers at various ratios was carried out. It is shown that as the percentage of monomer in the MMA-AA copolymer increases, the glass transition temperature shifts towards a lower temperature. According to the results of thermogravimetric analysis, it was found that the binders have high heat resistance and methacrylate bonding at a temperature of 300 °C and that the oxidation process proceeds in the range of 500 °C, showing that thermal oxidative degradation is very stable. In addition, the DSC results show that increasing the amount of MMA in the copolymer increases the ***T_g_*** of the binders. The acrylic coating is a topcoat, 0.75 mm thick, applied over the base coat. The topcoat was applied using the premium E-Sirne 25/M5 primer, which, compared to the primer and base coat, offers superior resistance to water, chemicals, and abrasion. Acrylic floor primers are often referred to as quick-drying paints. They are composed of reactive acrylate and methacrylate resins and are characterized by rapid polymerization, making them a unique type of paint. In other words, highly durable polymer floor coatings harden within twenty-nine minutes of application. Mechanical and chemical testing of such a floor can be conducted within two hours. Special powder hardeners enable rapid curing. The speed of the polymerization reaction is complemented by the frost resistance of the quick-drying floor—such a coating can be applied at temperatures down to −30 °C. It has been established that increasing the number of COOH groups in the acrylic film increases the density of the crosslinking agent in the floor paint and reduces weight loss. A study of the chemical resistance of the acrylic film revealed that the primer obtained from the MMA-AA copolymer using this binder is durable and of high quality.

## Figures and Tables

**Table 1:** Recipe for preparing primer.

Group	Syrups	Copolymer (Powder, gm)	MMA (Liquid, gm)
Group 1	Syp10/M1	10	90
	Syp10/M2	10	90
	Syp10/M3	10	90
Group 2	Syp15/M1	15	85
	Syp15/M2	15	85
	Syp15/M3	15	85
Group 3	Syp25/M1	25	75
	Syp25/M2	25	75
	Syp25/M3	25	75

**Table 2:** Formulation of floor for primer.

Group	Name of Mix	Syrups (gm)	Sand (gm)	Calcium Carbonate (gm)	BPO (gm)	DMPT (gm)	Pigment (gm)
Group 1	F-Syp10/M1	100	150	100	1	0.75	5
	F-Syp10/M2						
	F-Syp10/M3						
Group 2	F-Syp15/M1						
	F-Syp15/M2						
	F-Syp15/M3						
Group 3	F-Syp25/M1						
	F-Syp25/M2						
	F-Syp25/M3						

**Table 3:** NMR signals and structural formula of MMA-AA copolymer (^1^H).

Functional Group	Signals (ppm)	Description
–OCH_3_ (1, 2, 3, 4)	3.55–3.64	Methoxyl protons of the methyl methacrylate unit (–COOCH_3_)
–CH– (8, 9, 12)	2.02–2.12	The methine protons in the main unit of the polymer (–CH–C–)
–CH_2_– and COO–(5)	0.97–1.90	Methylene protons of the side chains and carboxyl groups of the basic skeleton
–CH_2_–, –CH_3_ (6, 7)	1.22–0.98	Alkyl protons (end groups or initiator residues)
–CH_3_ (10, 11)	0.83–0.85	Methyl protons of the side groups MMA (–C(CH_3_)–)

**Table 4:** NMR signals and structural formula of MMA-AA copolymer ^13^C.

Functional Group	Signals (ppm)	Description
–COO– (carbonyl carbon)	176.4–178.2	Signals of carboxyl groups of acrylic acid and methyl methacrylate esters.
–OCH_3_	51.2–51.8	Methoxyl carbons of the MMA unit.
–C– (Quaternary carbon at C=O)	44.4–44.9	It is characteristic of the methyl methacrylate unit after polymerization.
–CH–	31.4–31.6	The methyl carbon bound to C=CH_2_ in the AA monomer
–CH_2_–	22.4–30.9 18.1–18.3	Additional methylene fragments, probably from impurities or a chain transporter
–CH_3_	13.4–14.1	Methyl carbons of the methyl methacrylate unit.

**Table 5:** Thermal characteristics of copolymers of MMA-AA.

[MMA-AA] mol. %	Tg, °C ^a^	Tg, °C ^b^	Temperature Range, °C ^b^	Lost Mass ^b^ %	Residual Weight ^a^ %	PDT_max_ °C ^b^
10-90	97.24	100	29.49–198.71 198.71–366.18 366.18–900.85	17.53 59.57 9.075	82.47 22.96 13.89	320
50-50	102.28	100	29.70–287.32 287.32–593.15 593.15–899.24	32.50 35.14 8.7165	67.65 32.51 23.76	380
90-10	103.01	100	29.80–219.07 219.07–275.67 275.67–333.32 333.32–438.71 438.71–899.16	14.76 4.54 5.59 14.19 20.26	85.24 75.15 40.80	500

Note: ^a^—values obtained in the analysis of DSC. ^b^—values of the Fox equation determined on the basis of TGA.

**Table 6:** Molecular weight characteristics of copolymers MMA-AA.

Copolymer Composition. %		M_n_·10^5^	M_w_·10^5^	M_z_ 10^5^	M_w_/M_n_
MMA	AA				
90	10	2.70	5.37	8.90	1.99
80	20	2.48	5.79	10.1	2.33
70	30	2.84	5.49	8.84	1.93
50	50	3.35	4.67	6.24	1.39
30	70	2.54	4.72	7.63	1.86
20	80	2.18	4.85	9.01	2.22
10	90	2.35	4.99	8.51	2.12

**Table 7:** Mechanical properties of MMA-AA copolymers depending on the molar ratio of monomers.

Copolymer Composition MMA-AA %	Compressive Strength, MPa	Weight Loss (mg/cm^2^)	Water Absorption (mg)	Pot-Life (min)	Curing Time (min)
F-Syr10/M1	39.9	0.0038	0.0007	28	50
F-Syr10/M2	45.8	0.0028	0.0005	16	35
F-Syr10/M3	41.1	0.0035	0.00055	19	46
F-Syr15/M1	44.8	0.0023	0.0006	21	48
F-Syr15/M2	53.5	0.0016	0.00032	15	32
F-Syr15/M3	48.9	0.0018	0.0004	17	37
F-Syr25/M1	46.4	0.0015	0.0005	15	41
F-Syr25/M2	59.9	0.0005	0.00051	25	29
F-Syr25/M3	55.7	0.001	0.00025–0.0003	14	35

## Data Availability

The original contributions presented in this study are included in the article. Further inquiries can be directed to the corresponding authors.
